# An Updated View of the Intracellular Mechanisms Regulating Cross-Presentation

**DOI:** 10.3389/fimmu.2013.00401

**Published:** 2013-11-22

**Authors:** Priyanka Nair-Gupta, J. Magarian Blander

**Affiliations:** ^1^Department of Medicine, Immunology Institute, Graduate School of Biological Sciences, Icahn School of Medicine at Mount Sinai, New York, NY, USA; ^2^Tisch Cancer Institute, Icahn School of Medicine at Mount Sinai, New York, NY, USA

**Keywords:** cross-presentation, major histocompatibility complex class I, pattern recognition receptor, Toll-like receptor, vesicular traffic, phagosomes, endosomes, dendritic cells

## Abstract

Cross-presentation involves the presentation of peptides derived from internalized cargo on major histocompatibility complex class I molecules by dendritic cells, a process critical for tolerance and immunity. Detailed studies of the pathways mediating cross-presentation have revealed that this process takes place in a specialized subcellular compartment with a unique set of proteins. In this review, we focus on the recently appreciated role for intracellular vesicular traffic, which serves to equip compartments such as endosomes and phagosomes with the necessary apparatus for conducting the various steps of cross-presentation. We also consider how these pathways may integrate with inflammatory signals particularly from pattern recognition receptors that detect the presence of microbial components during infection. We discuss the consequences of such signals on initiating cross-presentation to stimulate adaptive CD8 T cell responses.

## Introduction

Classically, endogenous antigens such as proteins synthesized by virally infected cells or tumor cells, are presented on major histocompatibility class I (MHC I) molecules for detection by CD8 T cells. However, a seminal study by Bevan (1) showed that in animals immunized with fully allogeneic cells, cytotoxic CD8 T cell responses were seen specific for minor antigens from the graft that were presented on MHC I molecules of the host (1). This finding indicated that antigens from the transplanted cells could be internalized by antigen presenting cells (APC) of the host and presented on the host MHC I molecules. Bevan termed the activation of CD8 T cells to this process of antigen transfer as “cross-priming,” and later the actual process of antigen transfer was called “cross-presentation” (2). A once poorly defined phenomenon, cross-presentation is now considered to be a critical mechanism to mediate immune responses against infectious agents and tumors as well as to induce peripheral tolerance (3).

The importance of cross-presentation becomes apparent given the existence of several viruses that exhibit strict tissue tropisms such as papilloma virus where infection is mainly confined to epithelial cells in the skin barrier (4). Other examples for viruses that do not infect APC include encephalomyocarditis virus (EMCV) and semliki forest virus (SFV) (5). Additionally, some viruses such as herpes simplex virus (HSV), measles, retrovirus, canarypox virus, vaccinia virus, and lymphocytic choriomeningitis virus infect APC, but impair direct presentation of antigen (6–13). Additionally, cross-presentation has been demonstrated to play a critical role in mediating CD8 T cell immune responses against parasitic infections such as *Toxoplasma gondii* (14). Cross-priming has also been studied in the context of bacterial infections such as *Listeria monocytogenes* and *Mycobacterium tuberculosis*. In these infections, host defense is primarily mediated by dendritic cells (DC) that phagocytose infected apoptotic cells and mediate cross-priming, thus allowing for effective cytotoxic T lymphocytes (CTL) responses against the pathogens (15–17). Hence, cross-presentation allows for a mechanism through which the antigen can be presented by the APC without the need for direct infection.

Although other phagocytes have been reported to cross-present antigen, DC are considered to be the primary cross-presenting cell. The superior ability of DC to cross-present is largely attributed to their antigen processing capacity. Endocytic pathways in DC preserve and retain antigen epitopes via low lysosomal proteolysis and expression of protease inhibitors (18). This aspect makes sense when one considers that DC pick up antigen in the peripheral tissue and migrate for several hours-days (12–24 h for dermal DC and 3 days for Langerhans cells) reaching the lymph nodes (19). Thus, instead of being processed and degraded prematurely, retention of antigen would allow optimal cross-presentation for subsequent recognition by lymph node resident naïve CD8 T cells. However, DC subsets are heterogeneous in their ability to cross-present antigens. Subsets such as conventional splenic and lymph node resident CD8α^+^ DC, migratory DC populations such as lung and dermal CD103^+^ DC as well as monocyte-derived inflammatory DC excel at cross-presentation (20–24). It is still unclear why these DC subsets are specialized for cross-presentation. Interestingly, conventional CD8α^+^ DC as well migratory CD103^+^ DC populations appear to rely exclusively on Batf3 and IRF8 transcription factors for their development (25–28). Therefore, it is curious to ask if the ability to cross-present is developmentally controlled by the Batf3/IRF8 transcription programs that enable these subsets to have unique and specialized compartments geared toward cross-presentation. On the other hand, *in vitro* studies argue that the ability of splenic CD8α^+^ DC to cross-present antigen is induced as a subsequent step in maturation aided by cytokines such as granulocyte-macrophage colony-stimulating factor (GMCSF) or exposure to microbial products (29).

Recent studies in human DC have proposed the lymphoid and non-lymphoid resident BDCA3^+^ (CD141^+^) DC to be the human counterparts of the cross-presenting murine lymphoid CD8α^+^ DC and non-lymphoid CD103^+^ DC (30–34). The BDCA3^+^ DC subset is indeed an attractive candidate for the human homolog as it shares with the murine CD8α^+^ and CD103^+^ DC several cell surface markers, including DNGR1 and XCR1, transcription factors such as Batf3 and IRF8, along with excelling at *in vitro* cross-presentation assays. However, evidence also exists contradicting the superiority of the BDCA3^+^ DC subset at cross-presentation (35–37). Furthermore, patients harboring an autosomal dominant mutation in *IRF8* selectively lose BDCA-1^+^ DCs but not BDCA3^+^ DCs in the peripheral blood, indicating that BDCA3^+^ DC is at least not developmentally regulated in the same manner as murine cross-presenting subsets (38). Additionally although Batf3 deficiency impairs development of BDCA3^+^ DC *in vitro*, humanized mice reconstituted with Batf3 deficient progenitors still display sufficient and comparable numbers of BDCA3^+^ DCs (39). Thus, even though the human BDCA3^+^ DC subset appears to be functionally related to the murine CD8α^+^ and CD103^+^ DC, further studies are warranted to determine their developmental program and subsequent specialization for cross-presentation.

Several groups have also focused their efforts on revealing the intracellular pathways and molecular mechanisms mediating cross-presentation at steady state. Three major mechanisms have been determined. The phagosome-to-cytosol pathway involves escape of the antigen into the cytosol, possibly mediated by Sec61, followed by degradation by the proteasome and transport of peptides into the endoplasmic reticulum (ER) by the transporter associated with antigen presentation (TAP) to be loaded onto MHC I molecules (40, 41). The ER-phagosome fusion pathway involves fusion of the ER-Golgi intermediate compartment (ERGIC) with the phagosome, leading to the formation of a so called “ERgosome,” where ERGIC components such as TAP and components of the peptide loading complex are recruited directly to the phagosome. This recruitment is mediated through pairing of the ER SNARE Sec22b with the plasma membrane SNARE syntaxin-4, which is also found on phagosomes (42). In this model, the antigen still requires escape into the cytosol for proteasomal degradation and is then imported back to the “ERgosome” by TAP to be loaded onto MHC I molecules (43, 44). In contrast to the cytosolic pathways, the vacuolar pathway involves direct processing of the antigen within the phagosome by endocytic proteases such as cathepsins and subsequent loading of peptides onto MHC I molecules (45).

Some of the above pathways suggest that cross-presentation takes place in a specialized intracellular compartment. This compartment could be endosomes or phagosomes depending on the size of the exogenous antigen and mode of internalization. For instance, in the ERgosome model, cross-presentation takes place in a subcellular compartment bearing characteristics of both the ERGIC and the endosome/phagosome. In this review, we elaborate on the vesicular pathways that serve to bring various components of the cross-presentation machinery to such specialized intracellular compartments. We discuss the unique combination of proteins in these compartments that make it attractive for cross-presentation at steady state and how this compartment might be modified and further optimized for efficient cross-presentation in the scenario of infection. We also review evidence for regulation of cross-presentation during microbial stimulation and discuss if this process can still take place at steady state.

## Nature of the Cross-Presenting Compartment at Steady State

**Figure 1:**
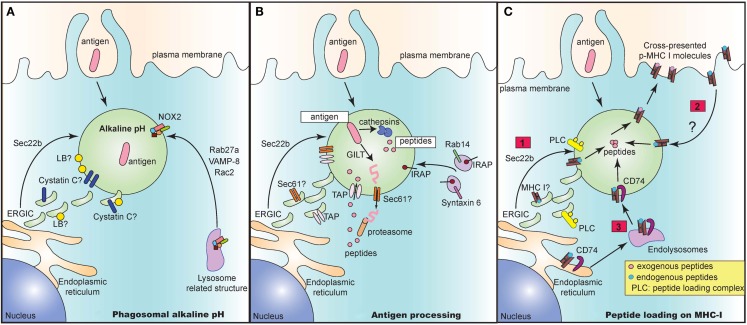
**The cross-presentation compartment at steady state**. Several vesicular pathways have been proposed to mediate delivery of key proteins that play an integral role in the crucial steps of cross-presentation including maintenance of alkaline pH **(A)**, processing of antigen to peptides **(B)** and subsequent loading of peptides onto MHC I molecules **(C)**. **(A)** Delivery of NOX2 via Rab27a, VAMP-8, and Rac2 is critical for maintaining an alkaline pH in intracellular cross-presenting compartments. Alternatively, recruitment of ERGIC via Sec22b also regulates pH and proteolytic activity in phagosomes. The ERGIC may contain protease inhibitors such as Cystatin C and lipid bodies (LB) that could directly alter phagosomal pH and enzymatic activity of proteases. **(B)** In the vacuolar pathway, antigen is directly processed into peptides by phagosome resident proteases such as cathepsins. In the cytosolic pathway, the antigen may need to be first unfolded by GILT prior to exit into the cytosol via a channel. This mystery translocon may be Sec61 and could be present in ERGIC, thereby recruited to the cross-presenting compartment via Sec22b. Once in the cytosol, antigen is then degraded into peptides and shuttled back into the phagosome via phagosomal TAP. TAP is dependent on Sec22b for its recruitment from ERGIC to endocytic compartments. Finally, IRAP, which is present on Rab14^+^ and Syntaxin 6^+^ endosomes, is recruited to phagosomes and mediates trimming of imported peptides that are further optimized for cross-presentation. **(C)** Lastly, processed and trimmed peptides have to now be loaded onto MHC I molecules. Source of MHC I in such compartments is unclear and could either be recruited from (1) ERGIC via Sec22b or (2) recycling from plasma membrane or (3) recruitment from endolysosomal compartments via CD74. In all cases, the peptide loading complex (PLC) is recruited from ERGIC via Sec22b to the compartment and can chaperone loading of exogenous foreign peptides to create “cross-presentable” peptide-MHC complexes.

In recent years, several groups have elucidated the architecture of the “cross-presenting” compartment at steady state. This compartment contains several identified proteins brought in by distinct vesicular pathways. These proteins may all be present in the compartment. Alternatively, these vesicular pathways may be mutually exclusive of one another, culminating in the presence of only some of these identified proteins. Regardless, these proteins serve as important players in executing different steps of the cross-presentation response (Figure 1).

### Maintenance of optimal alkaline pH

As antigens are internalized by endocytosis or phagocytosis, they undergo gradual proteolytic degradation along their journey from early endosomes and phagosomes to lysosomes (Figure 1A). Once in lysosomes, antigens are degraded by lysosomal proteases, which could destroy potential peptide epitopes crucial for T cell activation. DC circumvent this problem by expressing low levels of lysosomal proteases (18). Additionally, since most of these proteases function optimally at acidic pH (46), maintenance of a strongly alkaline pH in the cross-presentation compartment would inhibit protease activity, thus preventing overt and premature degradation of antigens. To this end, DC were reported to have high phagosomal pH, reaching values of 7.5–8 in contrast to macrophages which rapidly acidified their phagosomes, reaching values of 4.5–5 following phagocytosis of inert latex beads (47). Alkalinization of phagosomes in DC was attributed to selective recruitment, assembly and functioning of nicotinamide adenine dinucleotide phosphate (NADPH) oxidase NOX2. Finally, NOX2 activity was shown to be crucial for cross-presentation as genetic deletion of NOX2 subunit gp91phox led to abrogation of cross-presentation.

How does NOX2 reach the cross-presenting compartment? Recruitment of NOX2 is facilitated by Rab27a (48), a small guanosine tri-phosphatase (GTPase), which was initially characterized to mediate regulatory exocytosis of secretory vesicles in hematopoietic and non-hematopoietic cells (49). In a separate study, the Rac2 GTPase was demonstrated to also control the recruitment and assembly of phagosomal NOX2 in splenic CD8α^+^ DC as well as in *in vitro* derived bone-marrow derived DC (50). Additionally, VAMP-8, a SNARE protein which interacts with plasma membrane and phagosomal SNAREs syntaxin-4 and SNAP-23 (51), has also been recently reported to play a role in NOX2 recruitment and in mediating cross-presentation of phagocytic antigen (52). Interestingly, the protozoan *Leishmania* specifically cleaves VAMP-8 in phagocytes to prevent NOX2 assembly, thereby acidifying the phagosomes, in order to evade the cross-presentation response. However, it is still unclear if these GTPases and SNARE proteins act in concert or independently of one another to mediate recruitment of NOX2 and in turn to control cross-presentation. Given that VAMP-8 also participates in trafficking of secretory vesicles (53, 54), it is tempting to speculate that VAMP-8 and Rab27a might be present in similar secretory granules and are routed to the cross-presenting compartment upon entry of antigen.

In a second pathway, Sec22b mediated recruitment of ERGIC components has additionally been implicated in the maintenance of an alkaline pH (42). Sec22b silenced DC phagosomes have higher levels of mature cathepsin D, increased proteolytic activity, leading to accelerated degradation of antigen. These results therefore suggest that the ERGIC contains protease inhibitors. Which protease inhibitors could be involved? The cystatin family of protease inhibitors has been implicated to play a role in antigen presentation. Cystatin C was demonstrated to inhibit degradation of CD74, leading to enhanced accumulation of MHC II in endolysosomal compartments (31). Interestingly, cystatin C is abundantly expressed by CD8α^+^ DC compared to CD8α^−^ DC from the spleen (32), and only partially colocalizes with endolysosomal compartments (31, 32). Given that the cellular localization of cystatin C as well as its role in cross-presentation is still unclear, a feasible possibility is that cystatin C could perhaps colocalize with ERGIC and play a role in cross-presentation.

Another explanation for why recruitment of ERGIC would delay phagosome maturation is that the ERGIC may contain lipid bodies (LB) that have been implicated in regulating phagosomal alkalinization and antigen cross-presentation (55). These LB accumulate in the cytosol and on DC phagosomes in an interferon (IFN)-inducible ER-resident GTPase (Igtp) dependent manner. Specifically, Igtp was shown to interact with LB resident adipose differentiation related protein (ADFP) to mediate formation of LB, which were crucial for cross-presentation (55).

### Antigen processing

The cytosolic pathway model of cross-presentation stipulates that once antigen is internalized, it has to make its way out of the endosome/phagosome and into the cytosol for proteasomal degradation (Figure 1B). It is generally thought that prior to export into the cytosol, antigens may need to be unfolded. For certain antigens, this is a challenge owing to specific di-sulfide bonds holding the structure of the antigen together. In this case, gamma-interferon-inducible lysosomal thiolreductase (GILT) has been shown to be critical for cross-presentation of di-sulfide bonds containing antigen derived from HSV infected cells (56). Once unfolded, antigen is then routed to the cytosol through a channel, the identity of which still remains enigmatic and controversial. Sec61, a translocon involved in the ER associated degradation pathway (ERAD) was regarded as a top candidate given that blocking Sec61 activity by using *Pseudomonas aeruginosa* bacteria exotoxin A resulted in loss of cross-presentation of soluble OVA antigen (41). However, the evidence for exotoxin A directly and solely blocking Sec61 channel activity is still lacking. Interestingly, DC lacking Sec22b SNARE protein via short hairpin ribonucleic acid (shRNA) targeted deletion, showed impaired antigen export from endocytic compartments, thus arguing for the recruitment of an ERGIC resident translocon channel (42). Further studies analyzing phagosomal proteomics of these Sec22b sufficient and deficient DC would be integral to revealing the identity of the enigmatic translocon.

Once in the cytosol, it is well accepted that the antigen undergoes proteasomal degradation resulting in the generation of peptides. These peptides are then reimported by TAP into the lumen of the cross-presenting compartment (57). TAP is an ER and ERGIC resident protein that has been demonstrated to be recruited to phagosomes in a Sec22b dependent manner (42). Several groups have confirmed the dependence of cross-presentation on TAP, although in the case of certain bacterial antigens, cross-presentation can take place even in the absence of TAP via the vacuolar pathway where antigens are processed within endosomes and phagosomes by resident proteases (3).

Upon internalization of exogenous antigens, newly generated peptides can be trimmed by endosomal insulin-regulated aminopeptidase (IRAP) to be further optimized for cross-presentation (58). IRAP^−/−^ DC are impaired in their ability to cross-present soluble and particulate antigen, thus implicating the importance of IRAP in cross-presentation. IRAP colocalizes with Rab14^+^ and syntaxin 6^+^ endosomes at steady state, and is recruited to phagosomes after antigen uptake (58, 59). Whether IRAP depends on Rab14 GTPase and syntaxin 6 SNARE proteins for its delivery to the phagosomes remains to be studied.

### Peptide loading on MHC I

Processed and trimmed peptides are now faced with the possibility of being loaded on MHC I molecules that are present within endosomes and phagosomes (Figure 1C). A question that remains is where these MHC I molecules are recruited from. An attractive possibility is that MHC I molecules are present in the ERGIC and that perhaps Sec22b can deliver MHC I along with its chaperone proteins such as calreticulin and tapasin to the cross-presenting compartment. However, analysis of MHC I molecules in cell lines has revealed that MHC I molecules are transiently trafficked through the ERGIC at steady state. In fact, MHC I accumulated in ERGIC only in conditions where these molecules are not bound to high affinity peptides which could occur in the absence of TAP or calreticulin or when traffic out of the ERGIC is blocked (60–64). Whether MHC I trafficking in APC occurs similarly is still unclear.

An alternative possibility is that MHC I may be derived from the plasma membrane. Indeed, endocytosis and subsequent recycling of MHC I has been extensively documented in several cell lines where internalized MHC I are delivered to endosomal recycling compartments (ERC) in a step prior to being re-routed to the plasma membrane (63). Trafficking patterns of MHC I in APC are less clear. Some studies in APC confirm the reliance on plasma membrane derived MHC I, where internalization of surface MHC I molecules was shown to be dependent on a conserved tyrosine within the cytosolic domain of the MHC I, and to a lesser extent on a conserved serine phosphorylation site (65, 66). Mutations in these conserved sites resulted in inhibition of cross-presentation both *in vitro* and *in vivo*. However, given the strong evidence in cell lines for accumulation of MHC I in ERC, further studies are warranted to determine the contribution of MHC I molecules recycling through the ERC to cross-presentation.

Finally, it was also recently shown that CD74, which was originally characterized to route MHC II molecules from the ER to lysosomal compartments (67), was also important in mediating cross-presentation of viral and cell-associated antigen (68). CD74 was found in complex with immature endoglycosidase sensitive MHC I, indicating that it associates with newly synthesized MHC I in the ER (69). In CD74^−/−^ DC, MHC I molecules were present to a lesser extent in LAMP-1^+^ compartments, implying that CD74 delivers MHC I from the ER to endolysosomal compartments.

## Nature of the Cross-Presenting Compartment during Infection

In spite of these studies detailing the molecular and cellular makeup of cross-presenting compartments at steady state, the mechanisms underlying regulation and remodeling of this compartment during infection remain largely undefined. Upon uptake of microbial or infected cellular cargo, the phagosomal or endosomal compartment can be substantially modified by the acquisition of pattern recognition receptors (PRRs) (16). PRRs are evolutionarily conserved receptors that recognize and respond to conserved pathogen-associated molecular patterns (PAMPs) which are unique to microbes (70). Upon PRR engagement, intracellular signal transduction pathways are initiated such as those mediated by nuclear factor-κB (NF-κB), mitogen-activated protein kinases (MAPKs), and IFN-regulatory factors (IRFs) (71). These pathways are critical for providing immunity against several pathogen infections.

Several families of mammalian PRRs have been identified, namely Toll-like receptors (TLRs), nucleotide-binding oligomerization domain-like (NOD-like) receptors (NLRs), RIG-I like receptors (RLRs), and C-type lectin receptors (CLRs) (72). Indeed, TLRs were the first family of PRRs to be studied in detail (73–75). We focus here on recent developments in the biology of TLR that enable the recruitment of these receptors and assembly of their signaling machinery. This recruitment aids in the transduction of responses from the compartment itself, allowing localized phagosomal or endosomal specific control of responses, including presentation of exogenous antigen on MHC I and MHC II molecules. To signal and regulate presentation of exogenous antigen from endosomal compartments, TLRs first need to be recruited to the relevant compartment.

### TLR4

TLR4 is a plasma membrane resident “surface” TLR that can be endocytosed and also signal from intracellular compartments upon interaction with its ligand lipopolysaccharide (LPS). TLR4 initially engages toll-interleukin 1 receptor (TIR) domain-containing adaptor protein (TIRAP) and myeloid differentiation primary-response protein (MyD88) to initiate signal transduction from the plasma membrane (76). Subsequently, TLR4 is internalized into endocytic compartments and engages TRIF-related adaptor molecule (TRAM) and TIR-domain-containing adaptor inducing IFN-β (TRIF) (76). In fact, blocking TLR4 endocytosis using dynamin inhibitors selectively inhibits TRIF-TRAM mediated IRF3 dependent type I IFN responses without affecting TIRAP-MyD88 dependent signaling (77).

Recently, CD14 was shown to regulate the endocytosis of TLR4 from the plasma membrane during stimulation with LPS (78). TLR4 acts as a cargo for CD14, which transports the receptor and LPS to endosomes in a Syk and PLCγ2 dependent process where TRIF signaling leads to IFN-β production. While CD14 is critical for TLR4 endocytosis and IFN-β production in response to soluble LPS, TLR4 endocytosis in DC can proceed in the absence of CD14 during phagocytosis of *E. coli* or LPS-coated beads, although a lower percent of TLR4 is internalized compared to soluble LPS. Notably, despite lower percent TLR4 internalization in response to these particles, IFN-β production, which relies on TLR4 signaling from endocytic compartments, was unaffected in the absence of CD14. This result suggests that in the case of phagocytosed cargo, TLR4 can accumulate on phagosomes from another source independently of the plasma membrane.

Indeed, the small GTPase Rab11a was shown to play a crucial role in trafficking TLR4 from ERC to phagosomes containing gram-negative and not gram-positive bacteria, leading to IRF3 activation and IFN-β transcription (79). Additionally, adaptor protein 3 (AP-3) also plays a role in recruiting TLR4 and MyD88 from intracellular stores to phagosomes containing TLR4 ligands (80). Interestingly, AP-3 dependent recruitment of TLR4 and MyD88 was crucial for mediating production of pro-inflammatory cytokines selectively in response to phagocytic cargo and not to soluble LPS. Hence, the mode of uptake can dictate the pathway of delivery of TLR4.

It is curious to ask if the recruitment of TLR4 via these regulatory transport proteins contributes toward antigen presentation and adaptive immunity. For MHC II presentation, phagosome autonomous TLR4 signaling led to accelerated phagosome maturation and subsequent degradation of CD74 specifically in phagosomes bearing TLR ligands and not other phagosomes in the same DC (81, 82). Additionally, impaired recruitment of TLR4 to phagosomes in AP-3 deficient mice also led to decreased MHC II presentation (80). However, whether any of these proteins regulate cross-presentation of antigen internalized via endocytosis or phagocytosis remains to be investigated.

### TLR9

TLR9 is an endosomal receptor and begins its journey in the ER, where it associates with the chaperone protein Unc-93 homolog B (UNC93B), which mediates its transport to endosomes (83). Recently, recruitment of TLR9 and UNC93B was demonstrated selectively to phagosomes that contained DNA and anti-DNA immunoglobulin (Ig) complexes (84). Importantly, phagosomal TLR9 recruitment did not depend on its ability to sense the presence of TLR9 ligand DNA but instead relied on Fc receptor γ (FcRγ) mediated engagement by Ig complexes. These data suggest that Ig mediated FcRγ signaling leads to recruitment of TLR9 to phagosomes. When these phagosomes also contain the TLR9 ligand DNA, TLR9 signaling is engaged, resulting in IFN-α secretion. Given that engagement of FcRγ prepares phagosomes for optimal TLR9 signaling, it is tempting to speculate that this synergy between FcRγ and TLR9 may also impact subsequent cross-presentation responses of antigen complexed with DNA-Ig aggregates.

### TLR2

TLR2 is a cell surface TLR that synergizes with other surface TLRs such as TLR1 and TLR6 to mediate MyD88 dependent signal transduction responses. Similar to TLR4, there are reports showing TLR2 localization to endosomal compartments specifically early endosomes, lysosomes, and Rab11a^+^ vesicles in monocytes (85). Some studies also indicate TLR2 dependent induction of type I IFN signaling from endocytic compartments (86, 87). How TLR2 is directed to such compartments is still unclear.

## PRR Regulation of Cross-Presentation

Studies looking at the role of PRRs in cross-presentation have been largely limited to TLRs and CLRs. These receptors are well suited to regulate cross-presentation as they are present at the plasma membrane as well as along the endocytic pathway, where they encounter microbial antigen and initiate signaling to regulate adaptive immune responses such as cross-presentation. Here, we present evidence supporting regulation by these receptors.

### Toll-like receptors

There are several studies that show that TLR signaling enhances cross-priming of CD8 T cells (88). The *in vivo* contribution of TLR3 to cross-priming became clear after an elegant study demonstrated that signaling via TLR3 leads to maturation of DC and therefore promotes virus-specific CD8 T cell responses (5). Another study showed that injecting mice with apoptotic vesicles derived from *M. tuberculosis* infected macrophages, activates DC via TLR2 in a MyD88 dependent manner and can cross-prime CD8 T cells, thereby protecting mice from developing tuberculosis infection (17). Moreover, by employing biodegradable microspheres for the delivery of phagocytic cargo to DC, Schlosser et al. were able to demonstrate that the presence of both TLR ligand and antigen within the same phagosome yielded efficient CTL responses as compared to when the ligand and antigen are located in separate phagosomes (89).

One caveat of the studies above is the inability to distinguish the effects of TLR signaling on cross-presentation versus cross-priming. For example, a couple of studies revealed that TLR3 and TLR9 ligands could both induce cross-presentation of OVA by DC (90, 91). This induction of cross-presentation was found to be dependent on TLR signaling as DC from *Tlr9*^−^*^/^*^−^ and *Myd88*^−^*^/^*^−^ mice were unable to cross-present antigen after exposure to TLR ligands. However, the authors of these studies relied exclusively on T cell activation as a measure of displayed peptide-MHC I complexes. Given that TLR signaling also controls DC maturation and expression of co-stimulatory molecules that are pivotal for T cell activation, it is difficult to rule out the confounding factor of impaired co-stimulation and decreased DC maturation seen with TLR deficient DC. In fact, the inability of *Myd88*^−^*^/^*^−^DC to activate CD8 T cells after phagocytosis of virally infected cells was fully restored by treatment with a CD40 cross-linking antibody suggesting that defective cross-priming was due to impaired TLR induced co-stimulation rather than cross-presentation *per se* in this particular case (92).

The most direct way to assess cross-presentation is to use a conformation dependent antibody directly against preformed peptide-MHC I complexes on the surface of the APC. However, these antibodies are quite insensitive and work successfully only when DC were pulsed with large amounts of antigen. Nevertheless, Christian Kurts’ group successfully used 25D1.16 antibody to detect SIINFEKL-MHC I complexes and thus demonstrated increased cross-presentation with TLR signaling after uptake of soluble OVA in the presence of LPS (93). This increase in cross-presentation was mediated by TLR4, MyD88 and not TRIF signaling. However, this study was focused on the cross-presentation of soluble antigen and hence whether TLR signaling enhances cross-presentation of phagocytosed particulate antigen remains to be determined.

### C-type lectin receptors

C-type lectin receptors contain at least one carbohydrate recognition domain via which they bind to sugar moieties on self or microbial derived antigens. CLRs can regulate cross-presentation, although most of them do so by regulating antigen uptake. For example, CD209 and mannose receptor have been reported to increase internalization of cargo and target antigens to early endosomes for cross-presentation (94, 95). DNGR1 was also reported to enhance cross-presentation of cellular antigens derived from necrotic dying cells (96). Interestingly, despite intact maturation phenotypes and maintenance of signals to relay co-stimulation in DNGR1 deficient DC, these DC were impaired in cross-presentation, suggesting specific regulation of cross-presentation rather than cross-priming (97). DNGR1 is also expressed at high levels in subsets of DC specialized for cross-presentation including murine CD8α^+^ DCs and tissue-resident CD103^+^ CD11b^−^ DCs as well as in human counterparts BDCA3^+^ DC (39).

## Can Cross-Presentation Still Take Place at Steady State?

In the absence of inflammation or infection, cross-presentation of self-antigens at steady state can take place, leading to tolerance to host antigens and deletion of potentially auto-reactive CD8 T cells. Indeed, generation and activity of CTL must be tightly controlled to avoid auto-reactivity to self, given the potency of CTL in killing infected target host cells (98, 99). Here, we review existing evidence for the role of cross-presentation in both central and peripheral tolerance mechanisms.

### Peripheral tolerance

Peripheral tolerance constitutes mechanisms of tolerance that take place after mature lymphocytes enter into the periphery. There are several studies that argue for the constitutive TLR or PRR-independent nature of cross-presentation for the induction of peripheral cross-tolerance to non-inflammatory self-antigens, leading to deletion of self-reactive CTL. Many of these models employed the expression of neo-self-antigens under the control of tissue-specific promoters like the rat insulin promoter (RIP). These models ensure that the antigens are expressed outside of the thymus, allowing researchers to specifically study peripheral tolerance.

Cross-tolerance was first demonstrated when OVA specific OT-I CD8 T cells were efficiently deleted after being adoptively transferred into a mouse expressing OVA under control of the RIP (RIP-mOVA) (100). Cross-tolerance was also shown to be important for the control of endogenous auto-reactive CD8 T cells specific for naturally expressed self-antigen (101). In this study, the authors bred the RIP-mOVA mice with mice lacking GTPase Rac1 in CD11c^+^ cells to generate Rac1-RIP mice. Conveniently, deficiency in Rac1 GTPase selectively affected the ability of CD8α^+^ DC to internalize antigen (33), resulting in impaired cross-presentation while leaving the classical MHC II and MHC I pathways of antigen presentation unaffected (101). Consequently, they were able to demonstrate that DC in Rac1-RIP mice failed to cross-present transgenic self-antigen and hence failed to delete transferred OT-I T cells. Moreover these mice developed symptoms of diabetes. Interestingly, mice that just had Rac1 deleted in CD11c^+^ cells also had higher numbers of endogenous CD8 T cells, although the mice seemed healthy and did not develop autoimmunity. However, when CD25 depleted T cells from these mice were transferred into lymphopenic hosts, the hosts developed several signs of autoimmunity as a result of homeostatic T cell proliferation. Hence, the above studies clearly demonstrate the role of cross-presentation under steady state to induce peripheral tolerance.

An interesting study by Christian Kurts’ group looked to see if tolerogenic DC could be converted into autoimmunogenic DC after exposure to stimulating conditions such as TLR ligands (102). TLR ligands were able to induce CTL mediated autoimmunity only in cases where antigen specific CD4 T cell help was provided concomitantly. These results demonstrated that the mere presence of TLR ligands, such as those present in commensal bacteria or those derived from the use of vaccine adjuvants, is not sufficient to break cross-tolerance mechanisms.

### Central tolerance

In addition to peripheral tolerance, cross-presentation was also implicated in the induction of central tolerance (103). Central tolerance is induced in the thymus where developing thymocytes that recognize peptide-MHC complexes are positively selected to express either CD4 or CD8 molecules. Subsequently, thymocytes that are able to recognize self-peptide-MHC complexes with high affinity are efficiently deleted via negative selection. Medullary thymic epithelial cells (mTECs) and DC play a critical role in mediating negative selection. mTECs exclusively express a broad range of tissue-specific antigens (TSA) (104, 105). In spite of expressing both MHC II and MHC I molecules, mTECs are poor APC. Bevan’s group was the first to show that bone-marrow derived cells in the thymus were capable of cross-presenting antigen captured from mTECs (103). However, this study also showed that mTECs, by themselves, were capable of direct presentation on MHC I molecules and were sufficient to induce CD8 T cell deletion, thus diminishing the importance of cross-presentation in central tolerance. A recent report does point to the relevance of cross-presentation by human thymic DC. Upon analyzing peptides eluted from both MHC I and MHC II molecules of human thymic DC, the authors observed that around 22% of the MHC I ligands were derived from proteins present in the vesicular/extracellular compartment the presentation of which would typically be associated with the classical MHC II pathway (30).

## Conclusion

The studies we reviewed here certainly point to the constitutive nature of cross-presentation, however, an increasingly large body of work now provides strong evidence for the capacity to enhance cross-presentation by signals from inflammatory PRRs. Having mechanisms of regulation in place allows for the generation of robust CTL responses during an infection while maintaining induction of tolerance at steady state. Further insight into these regulatory mechanisms may potentially help in tailoring better therapeutic strategies to combat infectious agents as well as tumors, while preventing autoimmunity. Hence, elucidating the mechanistic differences in vesicular trafficking between steady state and inflammatory cross-presentation would be important for developing new rationales in the design of safe and effective vaccines for anti-viral, anti-bacterial as well as anti-tumor immunity.

## Conflict of Interest Statement

The authors declare that the research was conducted in the absence of any commercial or financial relationships that could be construed as a potential conflict of interest.
